# Characteristics of Fecal and Mucosa-Associated Microbiota in Chinese Patients With Inflammatory Bowel Disease

**DOI:** 10.1097/MD.0000000000000051

**Published:** 2014-08-04

**Authors:** Liping Chen, Wei Wang, Rui Zhou, Siew C. Ng, Jin Li, Meifang Huang, Feng Zhou, Xin Wang, Bo Shen, Michael A. Kamm, Kaichun Wu, Bing Xia

**Affiliations:** Department of Gastroenterology (LC, WW, RZ, JL, MH, FZ, BX), Zhongnan Hospital of Wuhan Univeristy; Hubei Clinical Center and Key Laboratory of Intestinal and Colorectal Disease (BX), Wuhan; Department of Medicine and Therapeutics (SCN), Li Ka Shing Institute of Health Science, Chinese University of Hong Kong, Hong Kong; Department of Gastroenterology (XW, KW), Xijing Hospital of the Fourth Military Medical University, Xi’an, China; Department of Gastroenterology/Hepatology (BS), The Cleveland Clinic Foundation, Cleveland, Ohio, USA; and Department of Gastroenterology (MAK), St Vincent’s Hospital, Melbourne, Australia.

## Abstract

The intestinal microbiota plays an important role in the pathogenesis of inflammatory bowel disease (IBD), and geographical and genetic backgrounds impact the composition of the intestinal microbiota. However, there is a lack of evidence regarding the overall changes and characteristics of fecal-associated microbiota (FAM) and mucosa-associated microbiota (MAM) in Chinese patients with IBD. We recruited 26 patients with Crohn’s disease (CD), 46 patients with ulcerative colitis (UC), and 21 healthy individuals; we collected matched fresh fecal and mucosal samples from the same subjects. The microbial communities were studied by 454-pyrosequencing. Community-wide changes in FAM and MAM were observed in patients with IBD. The proportion of several butyrate-producing bacteria, such as of the genera *Roseburia*, *Coprococcus*, and *Ruminococcus* were significantly reduced, whereas the pathogens *Escherichia-Shigella* and *Enterococcus* were prevalent in patients with IBD. FAM and MAM were similar between CD and UC. FAM differed from MAM in healthy individuals and patients with UC. In conclusion, the compositions of FAM and MAM were altered in patients with IBD. The reduction of butyrate-producing bacteria and the increase in opportunistic pathogens might be associated with the pathogenesis of IBD.

## INTRODUCTION

Inflammatory bowel disease (IBD), which consists of Crohn’s disease (CD) and ulcerative colitis (UC), is a nonspecific chronic intestinal inflammatory disease of unknown etiology, and its incidence has been increasing in many developing countries over the last decades.^1,2^ It is generally thought that IBD occurs because of an imbalanced mucosal immune response to commensal bacteria in genetically susceptible individuals.^3^

Substantial data from experimental models and clinical studies have suggested that the gut microbiota plays an important role in the pathogenesis of IBD. The diversion of the fecal stream improves symptoms in patients with CD, and the postoperative exposure of the neo-terminal ileum to the luminal contents induces inflammation.^4,5^ Patients with CD may respond to antibiotic therapy.^6–9^ Serum antibodies against microbial antigens, such as anti-*Saccharomyces cerevisiae* antibodies, have been detected in IBD.^10^ In animal experiments, bacterial colonization can induce colitis in immune-deficient mice but not in undergerm-free conditions.^11,12^ Additionally, the transplantation of intestinal flora from immune-deficient mice with spontaneous colitis to normal mice can induce colitis.^13–15^

Ecological studies have indicated that patients with IBD have decreased mucosa-associated and fecal microbial diversity, reduced Firmicutes, *Clostridium coccoides*, *Bacteroides ovatus*, *B. vulgatus*, and **Faecalibacterium* prausnitzii* with increased Proteobacteria as well as a higher number of mucosa-associated aerobic and facultative anaerobic bacteria.^16–22^ Using a pyrosequencing technique, Willing et al^23^ found that populations of *Faecalibacterium* and *Roseburia* disappeared and that *Enterobacteriaceae* and *Ruminococcus gnavus* populations increased in patients with ileal CD.

The genetic background is not only associated with susceptibility to IBD but also impacts the community structure of the intestinal microflora^24,25^ and determines the host immune response to intestinal microflora.^26^Ethnic differences in IBD susceptibility genes have been reported.^27^ Thus far, the well-known NOD2/CARD15 gene accounts for up to 20% of CD in the Caucasian population; however, this gene is not associated with CD in the Japanese and Chinese populations.^28–30^

The overall characteristics of FAM and MAM in patients with IBD in the Chinese population have not been studied. In the present study, we used high-throughput pyrosequencing to study FAM and MAM in a cohort of Chinese IBD patients because of the advantages of pyrosequencing,^31^ and attempted to shed light on the role of intestinal microbiota in the pathogenesis of IBD.

## MATERIALS AND METHODS

### Study Population

This study was approved by the Ethics Committee of Zhongnan Hospital, Wuhan University, Wuhan, China (April 14, 2010), and informed consent was obtained from each subject before enrollment in the study. The diagnosis of IBD was based on clinical, endoscopic, radiological, and histological criteria.^32^ Patients with IBD who met any of the following criteria were excluded: use of antibiotics, probiotics, or prebiotics within the last 2 months; current infectious diarrhea; and use of infliximab within the last 6 weeks. Twenty-six patients with CD and 46 patients with UC were enrolled in the present study. UC activity was evaluated using the Sutherland Scores,^33^ and active UC was defined as an ulcerative colitis disease activity index >2. Activity of CD was scored best by Crohn’s disease activity index (CDAI),^34^ and active CD was defined as a CDAI > 150. Of the 26 patients with CD, 20 were active and 6 were quiescent. Of the 46 patients with UC, 40 were active and 6 were quiescent. Additionally, 21 healthy volunteers from Wuhan University were recruited as healthy controls. The demographic and clinical characteristics of patients with CD, patients with UC, and healthy individuals are shown in Table 1 (Tables S1–S3, http://links.lww.com/MD/A32, illustrate the demographic and clinical characteristics of healthy individuals and patients with UC and CD).

**TABLE 1:**
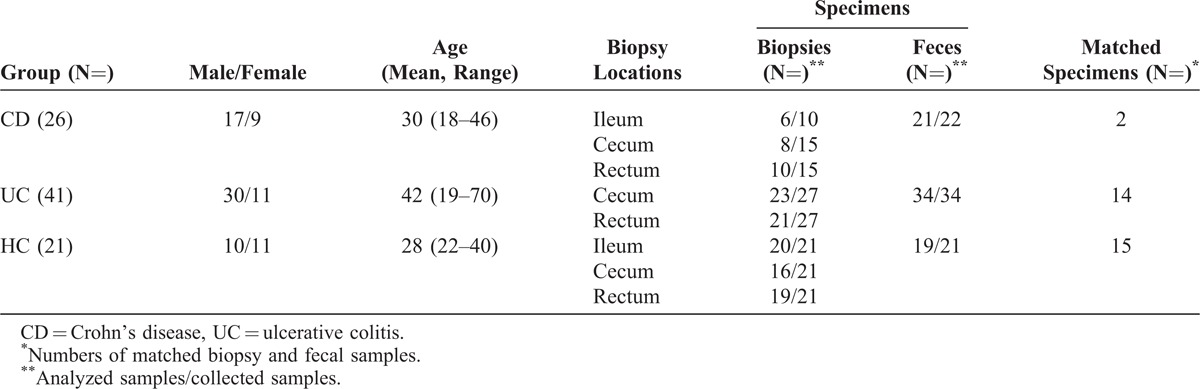
Specimen Numbers and Type of Enrolled Subjects

### Fecal and Mucosal Sampling

Fresh fecal samples were collected from 22 CD (CF), 34 UC (UF), and 21 healthy individuals (HF) and subsequently stored at −80°C in less than 1 hour to strictly prevent anaerobic bacteria from being exposed to oxygen and to avoid bacterial overgrowth before DNA extraction.

For mucosal sampling, colonic cleansing was performed using a 50% magnesium sulfate solution and water. Subsequently, colonoscopy was performed using a videoendoscope, and biopsies were obtained from 15 CD patients, 27 UC patients, and 21 HC individuals. The biopsies were obtained from the terminal ileum, cecum, and rectum in CD patients (CI, CC, and CR) and in healthy individuals (HI, HC, and HR) as well as from the cecum (UC) and rectum (UR) in UC patients using disposable forceps for each subject. Only location was considered, inflammation was not taken into account. The biopsies were sent to the laboratory on ice and were immediately stored at −80°C. The biopsies were not washed before storage.

Finally, we collected 77 fecal samples and a total of 157 biopsy samples. Ten patients with CD, 20 patients with UC, and 21 healthy individuals provided both mucosal and fecal samples. After the fecal matter was sampled, the colon was subsequently cleaned and colonoscopy was performed for examination. Biopsy specimens were then obtained from these subjects.

### DNA Extraction

DNA was extracted from 200 mg of feces, which was added to a 2-mL screw cap vial containing 300 mg of 0.1-mm glass beads (Sigma, St. Louis, Missouri) and was maintained on ice until the addition of 1.4 mL ASL buffer using the QIAamp DNA Stool Mini Kit (Qiagen, Hilden, Germany). The samples were immediately subjected to bead beating (45 s, speed 6.5) twice using a FastPrep-24 machine (MP Biomedicals, Solon, Ohio) before the initial incubation for heat and chemical lysis at 95°C for 5 minutes. Subsequent DNA extraction was performed following the QIAamp kit protocol for pathogen detection.

DNA was isolated from biopsy samples using the QIAamp DNA Mini Kit (Qiagen). Extraction was performed according to the manufacturer’s instructions, with an additional bead-beating step (45 s, speed 6.5, twice) using a FastPrep-24 machine at the beginning of the protocol. The extracted DNA was stored at −80°C until use.

### Pyrosequencing

Isolated fecal and mucosal DNA samples were used as templates for the amplification of the 16 S rRNA V1–V3 region by the barcoded broadly conserved primer 8 F and 533 R with the A and B sequencing adaptors. The forward primer (B-8F) was 5′-CCTATCCCCTGTGTGCCTTGGCAGTCTCAGAGAGTTTGATCCTGGCTCAG-3′, in which the sequence of the B adaptor is underlined. The reverse primer (A-533R) was 5′-CCATCTCATCCCTGCGTGTC TCCGACTCAGNNNNNNNNNNTTACCGCGGCTGCTGGCAC-3′, in which the sequence of the A adaptor is underlined, and the NNNNNNNN is designated as the unique 8-base barcode used for tagging each polymerase chain reaction (PCR) product. The length of the amplicon, including the 454 primer and the barcode, was 596 nt.

### Bioinformatic Analysis

Pyrosequencing reads produced by the above-mentioned criteria were simplified using the “unique.seqs” command to generate a unique set of sequences and were then aligned using the “align.seqs” command and compared using the Bacterial SILVA database (SILVA version 106; http://www.arb-silva.de/documentation/background/release-106/). Furthermore, the aligned sequences were trimmed, and the redundant reads were eliminated using the “screen.seqs,” “filter.seqs,” and “unique.seqs” commands in order. The “chimera.slayer” command was used to determine chimeric sequences. The “dist.seqs” command was performed, and unique sequences were clustered into operational taxonomic units (OTUs) defined by 97% similarity. In the present study, data preprocessing and OTU-based analysis were performed using Mothur, Version 1.24.0, http://www.mothur.org/wiki/Main_Page.

Unweighted Unifrac distance metrics analysis was performed using OTUs for each sample, and principal component analysis (PCA) was conducted according to the matrix of distance.

### Statistical Analysis

The Mann–Whitney test, Kruskal–Wallis test, and ANOVA were used to evaluate the differences in the bacterial populations between the CD, UC, and healthy individuals. The Mann–Whitney test, Kruskal–Wallis test, and ANOVA were conducted using SPSS version 17.0 for Windows (SPSS Inc, Chicago, Illinois).

## RESULTS

### Characteristics of Microbiota in Study and Control Groups

Pyrosequencing data for 3 fecal samples and 44 mucosal samples could not be obtained for unknown reasons; microbial DNA could not be amplified from these samples using the barcoded 454 primers. Finally, we obtained 180,101 tags for 74 (19 HF, 21 CF, and 34 UF) fecal samples (average, 2433 per sample), 78,565 tags for 26 (20 HI and 6 CI) terminal ileal mucosal samples (average, 3021 per sample), 106,928 tags for 47 (16 HC, 8 CC, and 23 UC) cecal mucosal samples (average, 2275 per sample), and 119,589 tags for 50 (19 HR, 10 CR, and 21 UR) rectal mucosa samples (average, 2392 per sample). The number of tag sequences remaining per sample ranged from 781 to 6165, and only 1 sample had fewer than 1000 tag sequences. The average sequence length was 500 bp.

The community richness (Chao 1 index) and diversity (Shannon index) were compared between the groups (Table 2). The Shannon index of group HF was significantly higher than those of group CF and group UF, and the Shannon index of group HC was significantly higher than those of group CC and group UC. Finally, the Shannon index of group HR was significantly higher than those of group CR and group UR (Table S4, http://links.lww.com/MD/A32, illustrates the detailed characteristics of each sample in the healthy individual group, UC patient group, and CD patient group).

**TABLE 2:**
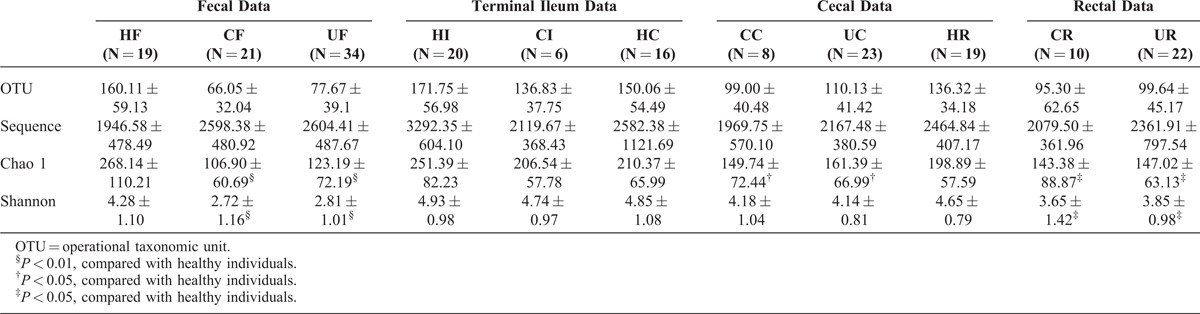
Pyrosequencing Data Summary

To determine whether all OTUs presented in the data set were recovered in this pyrosequencing study, rarefaction analysis was performed. The estimates were still increasing, even at the highest numbers of OTUs analyzed, which indicated that substantial unseen OTUs existed in the original samples and would only be detected after determining larger numbers of sequences (Figure S1, http://links.lww.com/MD/A32, illustrates the rarefaction curves of FAM and rectal MAM of CD patients, UC patients, and healthy individuals).

### Distinctive Fecal Microbial Communities in IBD

**FIGURE 1:**
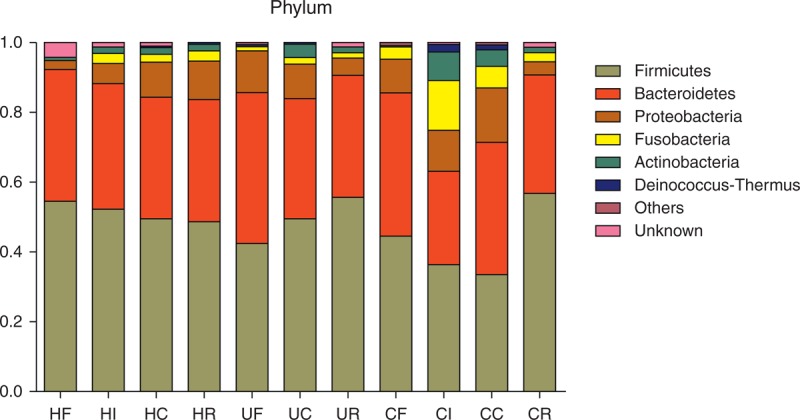
Relative abundance of primary bacterial phyla in different groups of samples. “Others” represents the Synergistetes, TM7, Tenericutes, Verrucomicrobia, Lentisphaerae, Acidobacteria, Gemmatimonadetes, Nitrospira, Planctomycetes, SR1, Spirochaetes, Armatimonadetes, Chloroflexi, and OD1. The first 5 phyla were not apparent in stool samples. “Unknown” represents the unclassified bacteria.

In fecal and mucosal samples, the dominant sequences belonged to 5 phyla that included Firmicutes, Bacteroidetes, Proteobacteria, Fusobacteria, and Actinobacteria (Figure 1).

**FIGURE 2:**
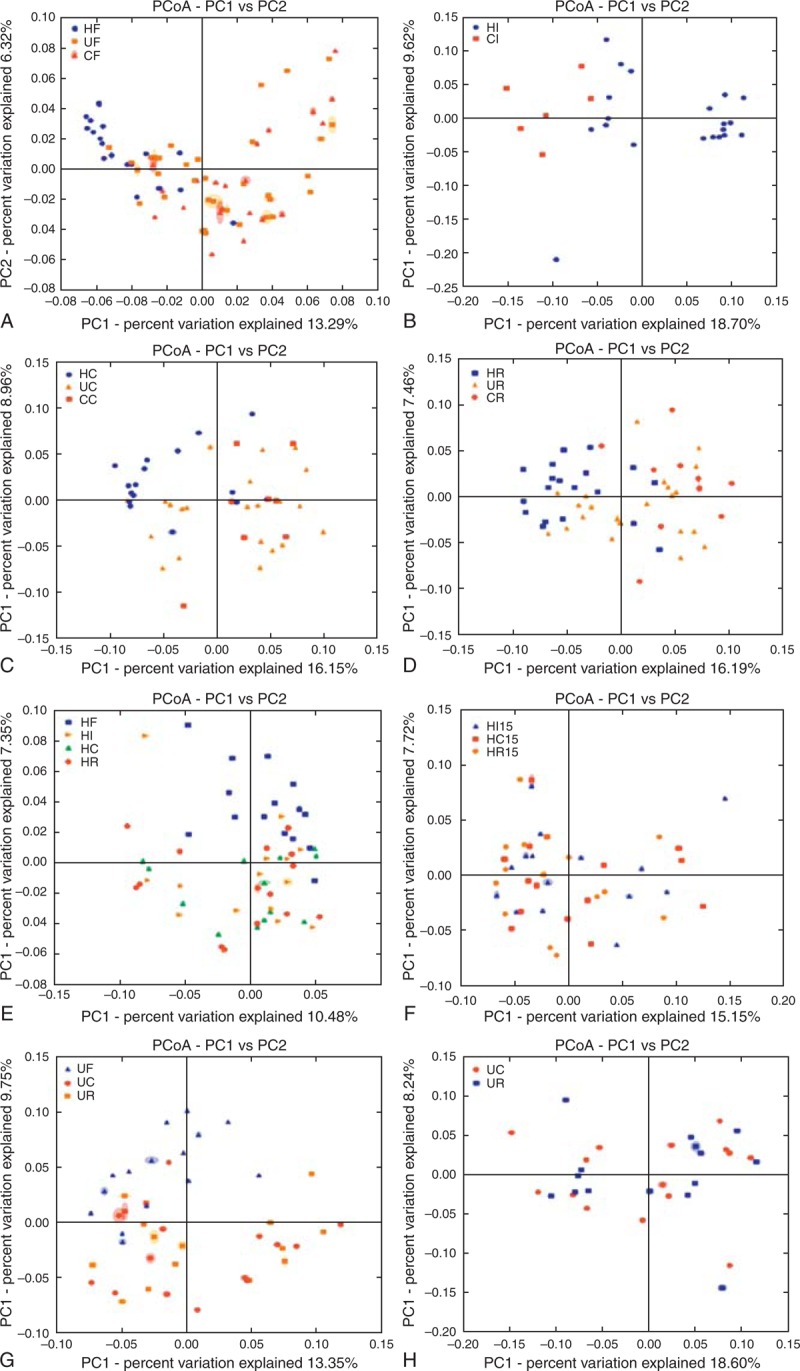
PCA plots based on unweighted Unifrac metrics. Each symbol represents a sample. (A) Groups HF, CF, and UF. (B) Groups HI and CI. (C) Groups HC, CC, and UC. (D) Groups HR, CR, and UR. (E) Groups HF, HI, HC, and HR. (F) Groups HI, HC, and HR. (G) Groups UF, UC, and UR. (H) Groups UC and UR.

To compare the overall microbiota structure among CF, UF, and HF, the unweighted Unifrac distance matrix was calculated based on the OTUs of each group. The results of PCA based on distance revealed a significant difference in the fecal microbial community between patients with IBD and healthy individuals; however, CF and UF overlapped and could not be well separated from PC1 and PC2 (13.29% and 6.32% of the explained variance, respectively) (Figure 2A).

There were significant differences in the composition of fecal microbiota at different taxa levels. Compared with HF, Fusobacteria were significantly higher in CF, UF demonstrated a marked decrease in the relative abundance of Synergistetes, and Proteobacteria were highly enriched in CF and UF. Only Synergistetes was significantly different between CF and UF.

As for CF, there were 5 significant differences at the family level compared with HF. The relative abundance of Streptococcaceae and Enterococcaceae were significantly higher than their abundances in HF. There was a significantly lower level of Ruminococcaceae, Rikenellaceae, and Acidaminococcaceae compared with HF. For UF, the relative abundance of Bacteroidaceae, Bacillaceae, Lactobacillaceae, Eubacteriaceae, Neisseriaceae, Enterobacteriaceae, Pasteurellaceae, and Enterococcaceae were significantly higher than that of HF. There was a significantly lower level of Actinomycetaceae, Carnobacteriaceae, Hyphomicrobiaceae, and Synergistaceae compared with HF. The abundance of Corynebacteriaceae, Lactobacillaceae, and Synergistaceae was significantly different between CF and UF.

The fecal microbial composition was also different at the genus level. There were 25 and 19 genera in CF and UF, respectively; these values were significantly different from HF. For several genera that constitute more than 1% of the total bacteria, *Streptococcus* and *Enterococcus* were highly enriched in CF. *Coprococcus*, *Roseburia*, *Faecalibacterium*, and *Ruminococcus* were significantly lower in CF compared with HF. *Bacteroides*, *Enterococcus*, *Blautia*, and *Escherichia-Shigella* were markedly enriched in UF. *Coprococcus* was significantly decreased in UF. The abundance of 7 genera was significantly different between CF and UF; however, no single genus exceeded 1% of the total bacteria (Table S5, http://links.lww.com/MD/A32, illustrates the detailed phylotypic differences in FAM among CF, UF, and HF).

### MAM in the Terminal Ileum of CD and Healthy Individuals

According to the unweighted Unifrac PCA analysis, the microbial communities of CI and HI were different from those of PC1 and PC2 (18.7% and 9.62% of explained variance, respectively) (Figure 2B).

A taxonomy-based comparison was performed to determine the microbiota differences between CI and HI. Proteobacteria were highly enriched in CI. There were 9 significant differences in CI compared with HI at the family level. Prevotellaceae, Peptostreptococcaceae, Ruminococcaceae, and Erysipelotrichaceae, which constitute more than 1% of total bacteria, were markedly lower in CI. Burkholderiaceae, which accounts for more than 1% of total bacteria, were highly enriched in CI. The genera *Prevotella*, *Coprococcus*, *Roseburia*, *Ruminococcus*, and *Sutterella* showed a significantly low abundance, and the genus *Delftia* was enriched in CI (Table S6, http://links.lww.com/MD/A32, illustrates the detailed phylotypic difference in MAM between CI and HI).

### MAM in the Cecum of CD, UC, and Healthy Individuals

The overall cecal microbiota structure of IBD was significantly different from that of healthy individuals; however, CC and UC overlapped and could not be separated from PC1 and PC2 according to unweighted Unifrac PCA analysis (16.15% and 8.96% of the explained variance, respectively) (Figure 2C).

For CC, families Rhodocyclaceae, Pasteurellaceae, Aeromonadaceae, and Carnobacteriaceae were highly enriched, and the abundance of family Prevotellaceae was significantly low in CC compared with HC. The genera *Prevotella*, *Coprococcus*, and *Blautia*, which account for more than 1% of the total bacteria, were markedly lower in CC. No genus that constitutes more than 1% of the total bacteria was highly enriched in CC.

In UC, the families Flavobacteriaceae, Enterococcaceae, Erythrobacteraceae, Sphingomonadaceae, and Alcaligenaceae had a higher abundance, and the family Prevotellaceae had a lower abundance compared with HC. For several genera that constitute more than 1% of the total bacteria, *Prevotella* and *Coprococcus* were markedly lower in UC; however, *Chryseobacterium* and *Enterococcus* were highly enriched in UC.

Compared with UC, Firmicutes was significantly lower in CC. The genera *Chryseobacterium* and *Blautia*, which account for more than 1% of the total bacteria, were decreased, and no single genus accounting for more than 1% of the total bacteria was increased in CC compared with UC (Table S7, http://links.lww.com/MD/A32, illustrates the detailed phylotype differences in MAM among CC, UC, and HC).

### MAM in the Rectum of CD, UC, and Healthy Individuals

Unweighted Unifrac PCA based on the OTUs in each sample revealed a separation between IBD patients and healthy individuals; however, CR and UR overlapped and could not be separated from PC1 and PC2 (16.19% and 7.46% of the explained variance, respectively) (Figure 2D).

In CR, the phyla Proteobacteria, Acidobacteria, Chloroflexi, and SR1 were highly enriched, while Firmicutes was reduced compared with HR. For several families and genera that constitute more than 1% of the total bacteria, Actinomycetaceae, Thermaceae, Bacillales_Incertae_Sedis XI, Enterococcaceae, Burkholderiales_Incertae_Sedis, and Enterobacteriaceae were enriched compared with HR. Prevotellaceae, Peptostreptococcaceae, and Ruminococcaceae were significantly lower than HR. The genera *Prevotella*, *Coprococcus*, *Roseburia*, *Dorea*, *Faecalibacterium*, *Ruminococcus*, *Megamonas*, and *Turicibacter* were markedly lower, and the genera *Actinomyces*, *Thermus*, *Tepidimonas*, *Escherichia-Shigella*, and *Enterococcus* were highly enriched in CR compared with HR. In UR, the microbial composition was not significantly different at the phylum level. Enterococcaceae had a higher abundance, and Prevotellaceae had a significantly lower abundance compared with HR. The genera *Prevotella*, *Turicibacter*, and *Ruminococcus* were markedly lower in UR, and the genera *Enterococcus* was highly enriched in UR.

There were several differences between CR and UR. Firmicutes was significantly lower in CR. The phyla Chloroflexi, Deinococcus-Thermus, Proteobacteria, and SR1 were significantly higher in CR. The abundance of families Thermaceae, Peptostreptococcaceae, and Ruminococcaceae that constitute more than 1% of the total bacteria exhibited significant differences between CR and UR. No genus that constitutes more than 1% of the total bacteria exhibited significant differences between CR and UR groups (Table S8, http://links.lww.com/MD/A32, illustrates the detailed phylotype difference in MAM among CR, UR, and HR).

### Matched FAM and MAM in Healthy Individuals and UC Patients

**FIGURE 3:**
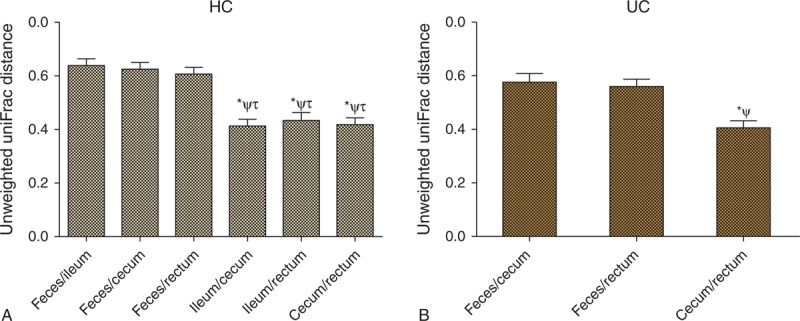
Unweighted Unifrac distance between matched feces and various mucosal samples from the same subject. (A) Unweighted Unifrac distance of healthy individuals. ^∗^*P* < 0.001, compared with the feces/ileum group; ^ψ^*P* < 0.001, compared with the feces/cecum group; ^τ^*P* < 0.001, compared with the feces/rectum group. (B) Unweighted Unifrac distance of UC patients. ^∗^*P* < 0.001, compared with the feces/cecum group; ^ψ^*P* < 0.001, compared with the feces/rectum group.

The overall microbiota structure differed between fecal samples and mucosal biopsies (Figure 2E and 2G). The mucosa-associated microbial composition overlapped and could not be separated from PC1 and PC2 according to the unweighted Unifrac PCA analysis in both healthy individuals and UC patients (Figure 2F and 2H). The unweighted Unifrac distances between various mucosal sites were significantly lower than that between feces and mucosa (Figure 3A and 3B).

As for healthy individuals, the abundance of Deinococcus-Thermus was significantly higher in various mucosal locations, Proteobacteria were markedly increased in the terminal ileum and cecum, and Fusobacteria were highly enriched in the terminal ileal mucosa compared with the feces. Only the abundance of Deinococcus-Thermus was different between the cecal and rectal mucosa.

For several families and genera that account for more than 1% of the total bacteria in healthy individuals, Erysipelotrichaceae and Comamonadaceae were highly enriched in mucosa biopsies. Ruminococcaceae was more prevalent in feces. *Faecalibacterium* and *Roseburia* were significantly increased in HF. *Dorea*, *Blautia*, and *Delftia* were highly enriched in the biopsies of healthy individuals.

As for UC patients, the abundance of Chloroflexi, Deinococcus-Thermus, Spirochaetes, and Synergistetes were significantly different between feces and mucosa. For some families and genera that constitute more than 1% of the total bacteria, Actinomycetaceae, Flavobacteriaceae, Erysipelotrichaceae, and Comamonadaceae were significantly enriched in mucosa biopsies. Ruminococcaceae was markedly increased in feces compared with the cecal biopsy. *Faecalibacterium* and *Parasutterella* were significantly more prevalent in feces than in the biopsies of UC patients. *Chryseobacterium*, *Actinomyces*, and *Delftia* were highly enriched in biopsies. No family or genus that constitutes more than 1% of total bacteria in biopsies exhibited significant differences in healthy individuals and UC patients. Detailed information is shown in Table S9 (http://links.lww.com/MD/A32), which illustrates the detailed phylotypes difference between every 2 groups of matched HF, HI, HC, and HR; Table S10 (http://links.lww.com/MD/A32) illustrates the detailed phylotype differences between every 2 groups of matched UF, UC, and UR).

## DISCUSSION AND CONCLUSIONS

Changes in the intestinal microbiota were common in patients with IBD.^16–22^ However, there was a lack of data on the overall changes of intestinal microbiota in Chinese patients with IBD. The incidence of IBD is increasing in China, and altered intestinal microbiota may contribute to the changing epidemiology. We used a 454-pyrosequencing technique to characterize the FAM and MAM in Chinese patients with IBD.

In our study, the diversity of fecal microbiota was significantly decreased in CD and UC patients. At the mucosa level, the diversity of the microbiota in the cecum and rectum of CD and UC patients was also significantly decreased, and the diversity of the microbiota in the terminal ileum of CD patients only tended to be lower compared with that of healthy individuals. These data are consistent with the previous studies.^20,35,36^ Reduced diversity of the intestinal microbiota can also be found in obesity, irritable bowel syndrome, *Clostridium difficile*-associated disease, acute diarrhea, and colorectal cancer, and reduced diversity of an infant’s intestinal microbiota is associated with an increased risk for developing allergic diseases at school age.^37–39^Until now, the reason for reduced bacterial diversity in IBD has not been established. It is hypothesized that the intestinal microbiota is not as diverse and evenly spread as a “rainforest” in healthy individuals; however, particular members may flourish under this specific condition.^40^

We analyzed the fecal and mucosal bacterial community of IBD and healthy individuals and found that the overall structure of the fecal and mucosal microbiota in patients with IBD significantly differed from that of healthy individuals. Additionally, our findings indicated that the overall compositions of FAM and MAM in CD patients were similar to those in UC patients and were not able to structurally separate according to unweighted Unifrac PCA analysis. In a study, Gophna et al^41^ found that MAM in CD patients was different from MAM in UC patients. In contrast, other studies suggested that the bacterial community structure was similar between CD and UC.^42,43^ The discrepancies between these results and ours may result from different analytical methods. Our work and other studies have focused on the comparison of the overall bacterial composition between CD and UC. However, Gophna et al^41^ only focused on the changes in a few types of bacteria between CD and UC. It is difficult to determine whether the shift of the intestinal microbiota is the cause or the result of IBD. The similarity of intestinal microbiota between CD and UC suggested that the alterations in bacterial composition might be the result of IBD.

Detailed compositional changes in intestinal microbiota in patients with IBD were investigated at different taxonomic levels. The genera *Roseburia*, *Coprococcus*, and *Ruminococcus*, affiliated with family Lachnospiraceae, were found to be significantly decreased in IBD. The genus *Faecalibacterium*, which belongs to the family Clostridiaceae, was markedly lower in the feces of CD patients. Lachnospiraceae participate in carbohydrate fermentation of short-chain fatty acids (SCFAs) in the human intestine.^44^ The health-promoting function of SCFAs includes roles as nutrients for the host and colonic epithelium and modulators of colonic pH.^45^ A decrease in SCFAs can result in damage to the normal function of the colonic epithelium. Previous studies have indicated that the abundance of *F. prausnitzii* is reduced in CD patients.^21^ Our study showed that fecal *Faecalibacterium* decreased in CD but not in UC. The data suggested that decreased levels of fecal *Faecalibacterium* were specific to CD.

The abundance of the genus *Escherichia-Shigella* within the Enterobacteriaceae family was increased in the feces of UC patients and rectal biopsies of CD patients. Considerable levels of *Gammaproteobacteria* have been observed under inflammatory conditions with *Salmonella*,^46^ which results in a Th1/Th17 immune response and mucosal damage similar to that observed in CD.^47^ We also found that the genus *Enterococcus*, which is affiliated with the *Enterococcaceae* family, was highly enriched in fecal and biopsy samples of IBD patients. Studies have suggested that *Enterococcus faecalis*, which is associated with intestinal inflammation, might play an important role in the pathogenesis of IBD because *E. faecalis* can induce IBD in IL-10 gene knockout mice.^48,49^The increased abundance of *Escherichia-Shigella* and *Enterococcus* indicated that they might be associated with the pathogenesis of IBD.

An accurate disease diagnosis is important for IBD. Recently, a pyrosequencing study in twins with IBD showed that ileal CD could be differentiated using a predictive model by partial least square discriminate analysis based on FAM.^23^ Additionally, pediatric patients with IBD can be distinguished from patients with similar IBD-related symptoms by FAM.^50^ Is MAM as valuable as FAM for the diagnosis of IBD? Controversy exists concerning the difference between FAM and MAM in healthy individuals or IBD patients.^51^ We investigated the differences between FAM and MAM. For healthy individuals and UC patients, our results indicated that FAM was significantly different from MAM according to an unweighted Unifrac PCA analysis, which suggests that IBD could be classified based on MAM. This opinion is supported by a recent study, which indicated that rectal MAM offers a unique potential for convenient and early diagnosis of CD compared with FAM.^52^

The primary limitation of this study was that mucosal DNA from a portion of the CD patients could not be amplified by the barcoded primer used in this research. We were unaware of the variation of MAM within the colon and the similarity between FAM and MAM in CD patients. Moreover, pyrosequencing results showed several differences compared with our previous q-PCR data.^53^ The reduced level of *Faecalibacterium* could be observed in stool and biopsy samples by q-PCR but was only observed in the stool samples of CD patients by pyrosequencing.^53^ A similar explanation for these differences might be that the specificity and sensitivity of primers used in our study were insufficient. A similar situation was reported by Chen et al.^54^ The results of pyrosequencing should be interpreted with caution.

In conclusion, our study presented a comprehensive view of the intestinal microbiota in Chinese patients with IBD using barcoded pyrosequencing. Our results demonstrated the following: the microbial composition in IBD was distinct from that of healthy individuals, the overall bacterial composition of CD was similar to that of UC, and FAM was different from MAM in UC patients and healthy individuals.
